# A mixed-methods study evaluating the impact of an excursion-based social group on quality of life of older adults

**DOI:** 10.1186/s12877-021-02295-7

**Published:** 2021-06-10

**Authors:** Joyce Siette, Mikaela Jorgensen, Amy Nguyen, Gilbert Knaggs, Stuart Miller, Johanna I. Westbrook

**Affiliations:** 1 Centre for Health Systems and Safety Research, Australian Institute of Health Innovation, Macquarie University, Macquarie, New South Wales 2109 Australia; 2 St Vincent’s Clinical School, University of New South Wales, Sydney, Australia; 3 Enrich Living Services, West Perth, Australia

**Keywords:** Wellbeing, Home and community care services, Social networks, Social capital

## Abstract

**Background:**

Social isolation is an increasing concern for older adults who live in the community. Despite some availability of social support programs to address social isolation, their effectiveness is not routinely measured. This study aimed to evaluate an innovative excursion-based program offering unique social experiences to older adults receiving aged care services.

**Methods:**

This six-month before and after mixed-methods study evaluated the outcomes of an Australian excursion-based program which offered social and physical outings to bring older adults receiving aged care services into the wider community. The study combined two parts: Part 1 was a pre-post survey assessing the quality of life of older adults who received the excursion-based program for 6 months (*n* = 56; two time-points, analysed using signed rank test) and Part 2 involved qualitative in-depth, semi-structured interviews (*n* = 24 aged care staff, older adults and carers; analysed using thematic analysis).

**Results:**

Older adults experienced a significant increase in quality of life scores (*p* < 0.001) between baseline and 6 months. Interviews confirmed these observations and suggested that benefits of participation included increased opportunities for social participation, psychological wellbeing, physical function, and carer respite. Interviews also revealed being in a group setting, having tailored, convenient and accessible activities, alongside supportive staff were key drivers in improving the wellbeing of participants.

**Conclusions:**

Participating in an excursion-based community program may improve wellbeing in older adults. Aging policy should focus on prioritizing initiatives that promote social connectivity with the wider community and assist in improving outcomes for older adults.

**Supplementary Information:**

The online version contains supplementary material available at 10.1186/s12877-021-02295-7.

## Background

People need social engagement and exchange to live fulfilled and enriched lives. Globally, older adults are at acute risk of becoming socially isolated as social networks and mobility levels decline with age [1, 2]. Consequences of social isolation are complex, with recent evidence suggesting that isolation takes a heavy toll on health [3], leading to poorer psychological and physical health outcomes [4].

Loneliness and poor health outcomes in older age are largely predicated on access to social capital [5, 6]. Social Capital Theory refers to the amount, type and nature of relationships – which may be institutional or more informal – that a person may draw on to obtain a sense of identity [7, 8]. Most older adults want to participate in their communities for as long as possible [9], however many lack the economic and social capital to do so [5]. From the early 1980s, in recognition of older people’s aspirations to age at home, the Australian Government incrementally introduced home care services and supports [10]. Today, Australian community-based care services act as a resource for older adults to remain in their own homes as they age, assisting older adults to manage or alleviate health risks associated with social isolation, immobility and aging in general [10]. These services include educational programs about health and wellness, opportunities for civic engagement, housework and hygiene assistance, as well as assistance with managing chronic health conditions [11].

Several systematic reviews have demonstrated the effectiveness of different community care and interventional programs in improving cognition, quality of life, and the physical and mental health of vulnerable older adults [4, 12–14]. Recent work has further identified the value of community-based social support services in delaying entry into residential aged care [15]. Community-based care services that can improve social connections in isolated older adults mostly focus on Adult Day Services (ADSs). ADSs usually have a psychosocial focus and allow older adults to interact with peers, become involved in and give back to their communities and live active lifestyles. Most ADSs targeting social isolation are centre based, operate on a ‘drop in’ basis and host culturally or gender specific activities for members. Other ADSs are decentralised and offer multiple accessible activities across public locations. In addition to providing a space for socialisation and exchange with peers, decentralised ADSs commonly promote interaction with the wider community [16]. Examples of decentralised activities include day trips and excursions, group horticultural therapies, and senior-friendly sporting events. Both centre based and decentralised ADS programs have been shown to positively influence older adults’ mental health by facilitating feelings of mutual acceptance and belonging [16–18].

Further evidence is required to increase our understanding of best practices and service delivery models that aim to increase social interaction among older adults. A recent systematic review on available social engagement programs found ADS programs to be promising, but there were major methodological weaknesses and inconsistencies between studies [16]. While ADSs have been shown to improve the psychological and physical wellness of older adults [16, 17], centre-based and decentralised ADSs are usually grouped as a generic category of aged care. Consequently, little is known about the effectiveness and the mechanisms driving the success of decentralised ADSs, including excursion-based programs.

The present study reports on an excursion-based social program accessed by older people living in Western Australia known as “Community Connections”. This mixed methods study aimed to: i) examine whether quality of life of participants of the Community Connections social program changed following 6 months involvement in the program; and ii) conduct an in-depth exploration of the experiences of participants involved in the social program.

## Methods

An exploratory mixed methods study was chosen to enable an in-depth exploration to determine whether participating in an excursion-based community social group (see Study Flow in Supplementary Figure 1) is associated with changes in quality of life.

Firstly, a pre-post study where participants completed a validated quality of life survey at two time points, baseline and 6 months after involvement in the social program, was carried out (Study Part 1). Following completion of the second survey data collection, the research team, who has extensive training in qualitative methods, then conducted semi-structured interviews with staff, participants and carers involved in the social program (Study Part 2). Synthesis of the results from the two methods was undertaken; specifically, the qualitative information obtained from the interviews was used to validate and expand the quantitative results obtained from the survey [19].

Data were collected in Perth, Australia between October 2018 to April 2019. This study received ethics approval (Reference 5201837916740) from the Human Research Ethics Committee at Macquarie University. Participants, data collection, and data analyses for both phases are summarised below.

### Setting and participants

#### Enrich living Services

Enrich Living Services is a home and community service provider for older adults based in Western Australia with over 600 staff and 1200 clients. The organisation was established to provide physical, social and recreational activities as well as educational and lifestyle support to older adults managing significant changes in their lives [20].

#### Inclusion/exclusion criteria

For the pre-post survey (Study Part 1), community-dwelling adults aged 65 years or older, who had attended at least one event as part of the social program offered by Enrich Living Services and able to complete a survey in English were eligible to participate.

For qualitative interviews (Study Part 2), eligible participants were community-dwelling older adults who had attended at least one event of the social program and were able to provide consent. Staff members who were involved in any aspect of the program (design, coordination or delivery) were eligible to partake in the qualitative interviews. Carers were eligible for the qualitative interviews if they were family members or friends of an older adult who had attended the social program for at least a month.

#### Intervention: community connections program

Enrich Living Services runs a Community Connections program which provides a range of activities for older people in the community. The purpose of Community Connections is to provide opportunities for older people to maintain positive social connections outside their homes through organised group day outings [20]. Outings typically comprise a broad range of activities, and were categorised into two categories: (1) highly adventurous experiences such as supported ice skating, Harley Davidson motorcycle rides, horse riding; (2) more regular social engagements such as shows (e.g., movies, theatre performances), river cruises, restaurant meals. Up to four different types of outings were offered each week and were provided to participants as a monthly roster. Popular activities, as determined by the participants, are repeated in the following month. Community Connections is unique in offering decentralised activities which take place exclusively in public and community settings. Activities range from 2 h to half a day and were run on a continuous basis as part of the service provision for Enrich Living Services clients.

#### Quality of life survey (study part 1)

Quality of life was assessed using the Adult Social Care Outcomes Toolkit (ASCOT) [21]. This survey measures social care-related quality of life in eight domains which cover basic aspects of social care-related quality of life (e.g. personal cleanliness and comfort) and higher order aspects (e.g. social participation), as well as a domain to measure how care impacts respondents’ self-esteem (e.g., dignity). All domains have 4 response options; the first response option represents the ideal situation and the last one represents the worst imaginable state. For example, the question for the ‘social participation and involvement’ domain is “Thinking about how much contact you’ve had with people you like, which of the following statements best describes your social situation? 1) I have as much social contact as I want with people I like; 2) I have adequate social contact with people; 3) I have some social contact with people, but not enough; 4) I have little social contact with people and feel socially isolated.

Developed by a combination of time trade-off and best-worse scaling, United Kingdom population values were used to weigh the items in accordance to their relative importance, which led to a final total score for each response ranging from − 0.171 to 1. Higher scores reflect greater well-being, with negative values accounting for care states considered worse than being dead. The ASCOT tool is particularly suited to client centred evaluations of aged care services, due to its usability and focus on daily life activities [22]. The tool has acceptable construct validity and concurrent validity [23].

Enrich Living Services’ aged care staff provided a blank copy of the ASCOT survey to participants, either during the outings or during routine client assessments, at two time points. The survey is used routinely within the organisation and staff are experienced in its delivery and usage. Surveys were typically distributed to participants to self-complete and were later entered into the provider’s records. If participants needed further clarification the staff and the research team was available to assist. The first survey was administered to respondents participating in the program, who were asked to complete the survey again after being in the social program for 6 months (completing both baseline and follow-up surveys). 110 clients completed the ASCOT at baseline and 56 completed the ASCOT at follow-up. Non-completers were categorised as refusals (*n* = 8, 14.8%), left the organisation (*n* = 6, 11.1%) and not contactable at time of follow-up (*n* = 40, 74.1%). Surveys were completed between October 2018 to March 2019. Participants who did and did not complete the survey at the second time point had no significant differences in age or gender.

Responses to the survey were entered into the electronic client management system of the aged care provider allowing the survey data to be linked with other information about each of the participants.

#### Sociodemographic and care program data

In Australia, the two major government subsidised community aged care programs are the Commonwealth Home Support Programme (CHSP) and the Home Care Package Program (HCP) [11]. CHSP provides entry level help for people requiring basic assistance with domestic chores, social engagement and transportation. HCP funding provides a greater level of support for older people with more complex health and social care needs. The Community Connections program was available for older adults who were receiving either CHSP or HCP funded-services. In addition to Community Connections, Enrich Living Services provides other community care services such as domestic assistance, personal care, gardening services and clinical care. Information about participants’ sociodemographic data such as age and gender were extracted from Enrich Living Services’ client management system and provided to the research team in a non-identifiable format. Information about the type of funding program was also extracted.

#### Semi-structured interviews (study part 2)

Enrich staff invited clients to participate in semi-structured interviews with the research team to gain insights into the Community Connections program. Twenty-four telephone interviews were conducted with participants who had been receiving aged care services from Enrich recently (e.g., length of time receiving services was 2 months) as well as longer-term (e.g., length of time receiving services was 12 years). To ensure a varied sample, the researchers interviewed both stakeholders who had recently commenced Community Connections, and others who had been involved in the program for some time (see Supplementary Table 1*).* This meant that participants in the pre-post study who were engaged in the program for a short period of time, as well as participants who have been involved over many years were invited. Interviewees consisted of Enrich community aged care staff (*n* = 10), participants of the social program (*n* = 11) and family carers of the participants who attended the social program (*n* = 3). A purposive sampling approach was used to select participants based on their demographic and geographical homogeneity. All participants agreed to participate in the interview and all interviews were conducted one-on-one, at a time available and convenient to the participant. Written consent was obtained prior to the interviews, followed by verbal consent on the day of the interview. Interview guides included open-ended questions to elicit interviewees’ descriptions of their experience of the social program (for clients), factors contributing to the success and failure of the program (for staff), and benefits of the program (for carers). Please see Supplementary Table 2 for the interview guide. Interviews ranged from 10 to 35 min and were conducted in March 2019.

### Data analysis

#### Quality of life survey (study part 1)

Descriptive information was calculated. Changes in quality of life (as measured by ASCOT total scores) between baseline and 6 months post involvement in the social program were analysed using a Wilcoxon Signed Ranks Test. Data normality of the ASCOT total score was tested with the Shapiro-Wilk test (*p* < 0.001), indicating a significant deviation from a normal distribution. Survey data were analysed using SAS 9.4 and SPSS V22.

#### Interviews (study part 2)

Interviews were audiotaped and professionally transcribed verbatim. Data were stored and analysed using NVivo. A thematic framework approach to analyse the data was adopted [24]. Familiarization began during the interview stage listening to participants responses and continued as audio recordings of the interviews were transcribed. The transcribing process, re-listening to the audio recordings and rechecking the transcripts contributed to the early interpretation of data and generation of themes. Transcripts were not returned to participants for commenting or correction. Themes began to emerge either directly from responses to themes embedded in the interview schedule or as new themes initiated by responses by participants. A sub-set of transcripts were initially coded by JS and AN to explore the emergent themes and the researchers devised a coding framework that was then applied to all transcripts until it was judged that no new information was acquired (see Supplementary Table 3). Charting further allowed an overall picture of the data to be developed as data was lifted from its original context and rearranged into themes and subthemes (see Supplementary Figure 2). The final stage of framework analysis involved searching for patterns, associations, concepts, and explanations in the data; and we looked for examples from the transcripts to illustrate elements of the themes identified. A second cycle of coding was conducted during which codes were grouped into themes and subthemes; verbatim quotes were identified to represent each theme and subtheme.

## Results

### Survey (study part 1)

Sociodemographic and service use characteristics of the 56 participants who completed the ASCOT survey at two points in time are reported in Table 1. The mean age of the participants was 80.8 years and 72.5% were female. These demographics are largely representative of users of community aged care services [25]. Of participants, 46.8% were widowed and 36.7% married. A majority of participants were using CHSP-funded (entry-level care) services (68%). Mean number of outings between baseline and follow-up survey was 13 (SD = 8).

**Table 1:** Characteristics of 56 older adults participating in the Community Connections program

	***N***	Percent of participants
**Age [SD]**	80.8 [5.8]	
**Gender**		
Female	37	72.5
Male	14	27.5
**Marital Status**		
Divorced	11	13.9
Married	29	36.7
Widowed	37	46.8
Single	2	2.5
**Funding type**^a^		
CHSP (entry-level care)	38	68.0
HCP (higher care)	18	32.0
**Mean outings**^b^ **[SD]**	13 [8.0]	–

^a^ Government funding type, Commonwealth Home Support Program (CHSP) or Home Care Package (HCP) are two options available for older adults

^b^ Mean number of outings between ASCOT assessments, range 2–38

#### Outcome measures

**Fig. 1:**
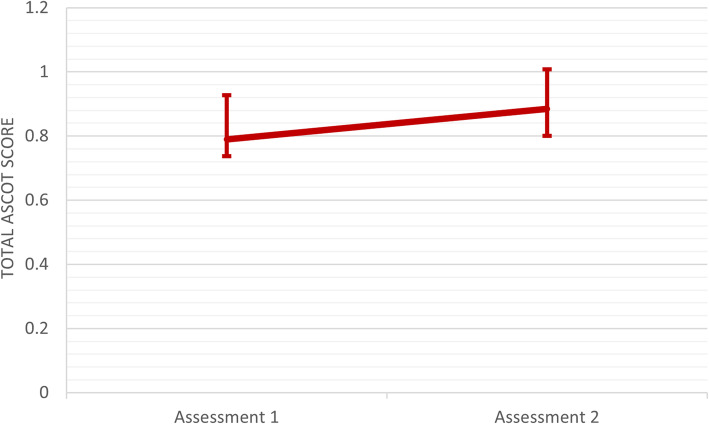
ASCOT scores at first and second time points for 56 older adults participating in the social program

Overall, participants reported moderate quality of life levels at baseline (Fig. 1). Social care-related quality of life increased significantly over time (Z = -4.772, *p* < 0.001) for the 56 participants. ASCOT total scores increased from a median of 0.74 at baseline (IQR = 0.65–0.91) to 0.89 (IQR = 0.83–0.97) 6 months later (Fig. 1).

Participants without follow-up scores (*n* = 54) had significantly higher baseline ASCOT total scores then those in the follow-up group (mean 0.88 (SD = 0.11) vs 0.74 (SD = 0.22), *p* < 0.001).

### Interviews (study part 2)

**Fig. 2:**
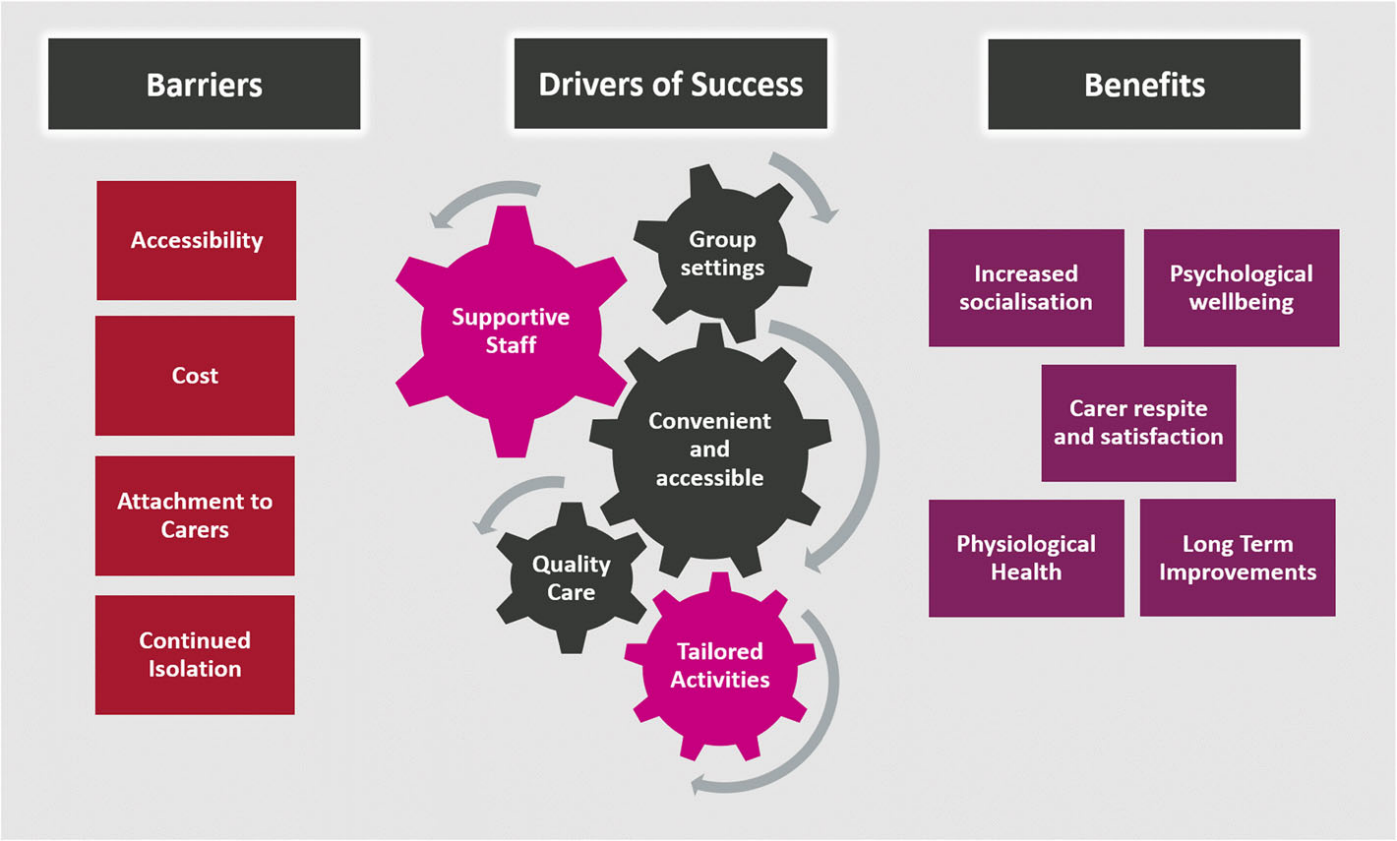
Themes and subthemes derived from the interviews. Original image produced by the authors

The mean age of clients was 83.2 years and 63.6% were female. The mean age of carers was 64 years and 66.6% were male. The mean age of staff was 48.2 years and staff had been with the company for an average 4.9 years. Participants evaluated the social program in almost exclusively positive terms. Analysis of the multiple stakeholder voices yielded three themes: a) *benefits associated with use,* including increased socialisation, psychological wellbeing, physical function, and carer respite; b) *drivers of success,* which included group settings, convenience, accessibility and supportive staff*;* and c) *barriers to use* (see Fig. 2).

#### Benefits

##### Increased socialisation

Almost all stakeholders reported that clients greatly enjoyed socializing with peers. Clients explained they established friendships which later developed into enduring relationships.

“We have got to know quite a few people. As a matter, I think, one of the girls … she’s getting married in Thailand in a couple of weeks’ time, and they want to come to our house here, in Perth.” (Participant 7)

Carers were similarly grateful that loved ones were provided opportunities to make friends, as finding time for such occasions had often otherwise proved difficult or impractical.

“Some old people, they never get out of their house, they never get a chance to meet others and we’ve made a lot of very good friends.” (Carer 1)

##### Improved psychological wellbeing

People interviewed explained that with age and declining health, mobility and social networks, clients’ everyday lives were lacking social interactions. Those using the program reported feeling happier and more confident. Some clients associated increased interactions with feeling like their younger selves.

“I sort of went into my shell for 10 years. And I wouldn’t go out, I didn’t go anywhere. … Now, I started going out with [Community Connections] and getting able to communicate with people again. And now I feel like my old self again.” (Participant 4)

Most carers noticed improvements in the psychological and social wellbeing of loved ones, reporting that overall those using Community Connections were happier and more engaged. However, some carers were uncertain whether change did occur due to the cognitive capacities of their family member.

“I mean she enjoys them, but she forgets everything the next day, so you wonder sometimes. The fact that she saw *Aladdin* twice, I often question, is there any point because she may enjoy it in the moment, but it doesn’t stay with her.” (Carer 3)

Carers also mentioned that having their family member attend outings improved their own quality of life, by providing them respite from caring duties and helping to break up the monotony of everyday life.

“I get a lot of respite, if we go out together, I can back off and they look after him, because otherwise I’m 24 hours a day carer for him.” (Carer 1)

##### Improved physiological wellbeing

Stakeholders explained that Community Connections influenced the physical health of clients. Clients spoke broadly of ‘getting out of the house’ as preferable to inactivity.

“They’re very – enjoyable all day. It gets me out of the house, because I’m going to get – I can’t be stuck in the house, I’ve got to get out somewhere.” (Participant 6).

Carers explained that Community Connections improved the mobility and fitness of users over time by encouraging clients to engage in greater amounts of physical activity than they would normally. Carers also believed increases in physical activity helped clients feel more confident and improved their psychological wellbeing.

“It’s just the joy that they reflect, the fact that they are not sitting at home watching some banal boring TV show that does not do anything for them. It means the physicality of getting out, it means moving around, it means stimulation of the mind, it means social interaction, it means the joy of actually being alive.” (Carer 2)

Staff also noticed that there were physical changes in clients over time as a result of joining Community Connections.

“They are physically getting stronger because they are out and about and walking. I’ve got people who were very, very fragile when we first started and needed hands-on assistance getting in and out of chairs, and now they’re fine. They’re fine, they get up and they get down, they take themselves to the toilet. It’s just the confidence and therefore their physical [capabilities] builds.” (Staff member 4)

##### Long term improvements

Stakeholders further described various lasting positive impacts. Clients most often discussed long-term social benefits of Community Connections, reporting that both the experience and anticipation of outings reduced feelings of loneliness at home.

“If you’re on your own you think, I won’t do that, I’ll do that tomorrow or I won’t do that. Now, when I go on outings, I’d be getting quite excited, ‘tomorrow we’re going out’ and it gives you that lift, you don’t feel down in the dumps.” (Participant 11)

Most carers explained that psychological and social improvements were ongoing and emphasised that they were noticeable within short periods of time.

“Dad went ice-skating yesterday. Came home and he was just grinning from ear to ear and this is someone who was highly depressive [ … ] So the behavioural changes are enormous.” (Carer 2)

Staff confirmed that relationships established during outings persisted. They further explained that day trips offered opportunities for initial social interactions which were further developed later by clients outside the context of Community Connections.

“They talk, they exchanged phone numbers, they meet on outings regularly, once a week they meet on an outing, and they have sleepovers at each other’s house.” (Staff member 4)

#### Drivers of success

##### Group setting

All stakeholders agreed that group settings were fundamental to the benefits received by clients. Clients cited the opportunity to engage in and complete recreational activities in a group setting as a primary driver of the benefits provided by Community Connections. For some, the program was understood more as an opportunity to socialise, rather than otherwise in-accessible recreational activities.

**“**I mean we could go to the pictures locally and things like that, but you’re going on your own, it’s not the same as having company.” (Participant 8)

##### Supportive staff

Clients were appreciative of staff for their professionalism but also approachability. Relationships formed with staff were integral to the benefits facilitated by involvement.

“I appreciate the carers coming… they are friendly, and check you, and hear what you’ve got to say. They probably take on some of our troubles as well. But they take it all in stride, and I formed quite a friendly sort of association.” (Participant 4)

##### Tailored activities

All stakeholder groups cited the ability of clients to book day options suited to their preferences as integral to success of Community Connections. For clients, the ability to choose from a range of activities suited both those who seeking to engage in specific and tailored activities, and others seeking an assortment of day trips.

“The outings are absolutely exceptional. They really are. The variety of places that they go to, the restaurants, the interaction. It would be 10 out of 10, couldn’t fault it in any way.” (Participant 2)

Staff also cited the range of activities to choose from as integral to Community Connections’ success. To ensure tailored care, staff routinely consulted clients for future day trips ideas and preferences.

“We will celebrate a special birthday, like 80 or 90, and book somewhere that that person wants to go. And then we advertise it in a calendar that this is [Client’s] 80th birthday, let’s go fly a helicopter, which is what she wanted to do.” (Staff member 4)

##### Convenient and accessible

All stakeholder groups mentioned accessibility and flexible transportation as key to the success of Community Connections. For many clients using Community Connections, venturing out of the house had become arduous. Clients greatly appreciated the provision of convenient, door-to-door transportation, which largely relieved difficulties and anxieties otherwise brought about by travel.

“The beauty of it is they pick you up, take you and bring you back home, and that’s 99% wonderful … you don’t have to worry about catching buses or trains, or driving anywhere.” (Participant 3)

Carers also noted the provision of transportation a contributing to the sense of relief afforded them by client involvement.

“It’s [transport] all done for you. It’s just such a luxury.” (Carer 3)

##### Quality of care

Clients were glad they were able to engage in social activities whilst still receiving more basic forms of care.

“They look after you. You only have to stand up and they say, ‘Where are you going?’ And you say you’re going to the toilet. They walk with you and make sure you’re all right and wait for you and bring you back to your seat.” (Client 5)

Carers also highlighted the importance of knowing loved ones were being well looked after and having their basic needs met during social outings.

“I can stay home, and he goes by himself, and there’s several carers that look after him. So, he’s not in any danger. They help him out of the car, they help him with his food, they help him with everything that he needs to do.” (Carer 1)

#### Barriers

##### Accessibility

Despite some stakeholders mentioning various barriers affecting program quality, most explained their appreciation of contextual factors inhibiting optimal service delivery. All stakeholder groups explained that it was becoming harder to access Community Connections due to the program’s expanding popularity.

“So many more people have come on board and when the sort of the page comes out of the outings and I phone them as soon as I get it and everything is all booked up and you’re put on a waiting list.” (Participant 2)

Carers also acknowledged they often had to book far in advance and identified that having an online booking system would be useful.

“As a future long-term suggestion, it would be really – but I understand the dynamics and the age group that we’re dealing with – it would be really helpful if we could book these outings online.” (Carer 2)

Staff spoke of wanting to expand the reach of Community Connections. In addition to growing waitlists, some staff hoped Community Connections could be made more accessible to older adults with higher care needs moving forward; although they also acknowledged that catering for such clients might not be financially viable.

“For those clients that have got real mobility issues, it’s a little bit more difficult and needs more planning, I suppose, to try and get those out, and, so that can be a bit more expensive.” (Staff member 5)

##### Cost

Staff identified several logistical barriers impacting the overall quality of Community Connections, most of which were associated with limited resources. A commonly discussed issue was funding, with concerns about sufficient staff numbers being a key point.

“I think the barrier, the major barrier would be funding, because it’s not cheap, it’s not cheap. You’re talking about, you’ve to pay staff, pay for the actual activity, you’ve got to pay for the transport. We’ve got to make sure all insurance are done, we’ve got to do research before implementing an outing …. All that costs money.” (Staff member 6)

##### Attachment to carers

Staff explained that some dependent clients had strong attachments to their family carers and were sometimes reluctant to participate without their support. However, staff encouraged family carers to be present on outings in such situations.

“We have had those clients that really do find it difficult to be separated from their loved one, and, so for that reason, we do try and get the carer to come along, and then, gradually, wean that carer away, so that the client’s happy.” (Staff member 5)

##### Continued isolation

Drivers of success identified by stakeholders did not uniformly produce increased socialisation and wellbeing in clients. Rather, some clients may continue to feel socially isolated during outings and require more care from staff. Of the clients interviewed one spoke of feeling socially disconnected and excluded from social interactions during outings.

“A couple of people gave me their phone number but there’s no – I don’t have any acquaintances … they’re chatting away and I look at it and it’s almost like, you know, you just want to cry because you just aren’t in that.” (Participant 2)

## Discussion

Our study presents evidence that excursion-based group community care programs can positively improve older adults’ quality of life. Our findings indicate that participation in this program was associated with a range of benefits related to increased socialisation and maintaining social connections, which can contribute to improved overall wellbeing.

Previous studies evaluating social activity groups targeting older adults have also shown improvements in wellbeing [26, 27]. However, such studies usually focus on older adults with specific physical and clinical needs in residential aged care [28, 29] or in the general population [30]. Descriptions of the social activities undertaken are also rarely described in any detail. Nevertheless, it is evident that long running community adult day services organisations with a similar structure to the Community Connections program (e.g., Seniors centres [31, 32], Men’s Sheds [33], University of the Third Age [34, 35], Japanese salons [36, 37]) are valuable in enabling social participation among older adults. This study adds to the evidence around the benefits of decentralised ADSs specifically and shows that this engagement may improve quality of life and can assist with reducing loneliness and maintaining social connections for older adults receiving community aged care.

Analysis of qualitative data revealed factors stakeholders felt were integral to benefits enjoyed by participants and their carers. These provide insights into why flexible, excursion-based care may be effective. All stakeholders regarded the social nature of the groups and increased opportunity for interaction as the most important feature of the program. Previous systematic reviews of ADS programs and interventions targeting social isolation have similarly identified social engagement elements and group settings as drivers of success [1, 16, 38, 39].

**Fig. 3:**
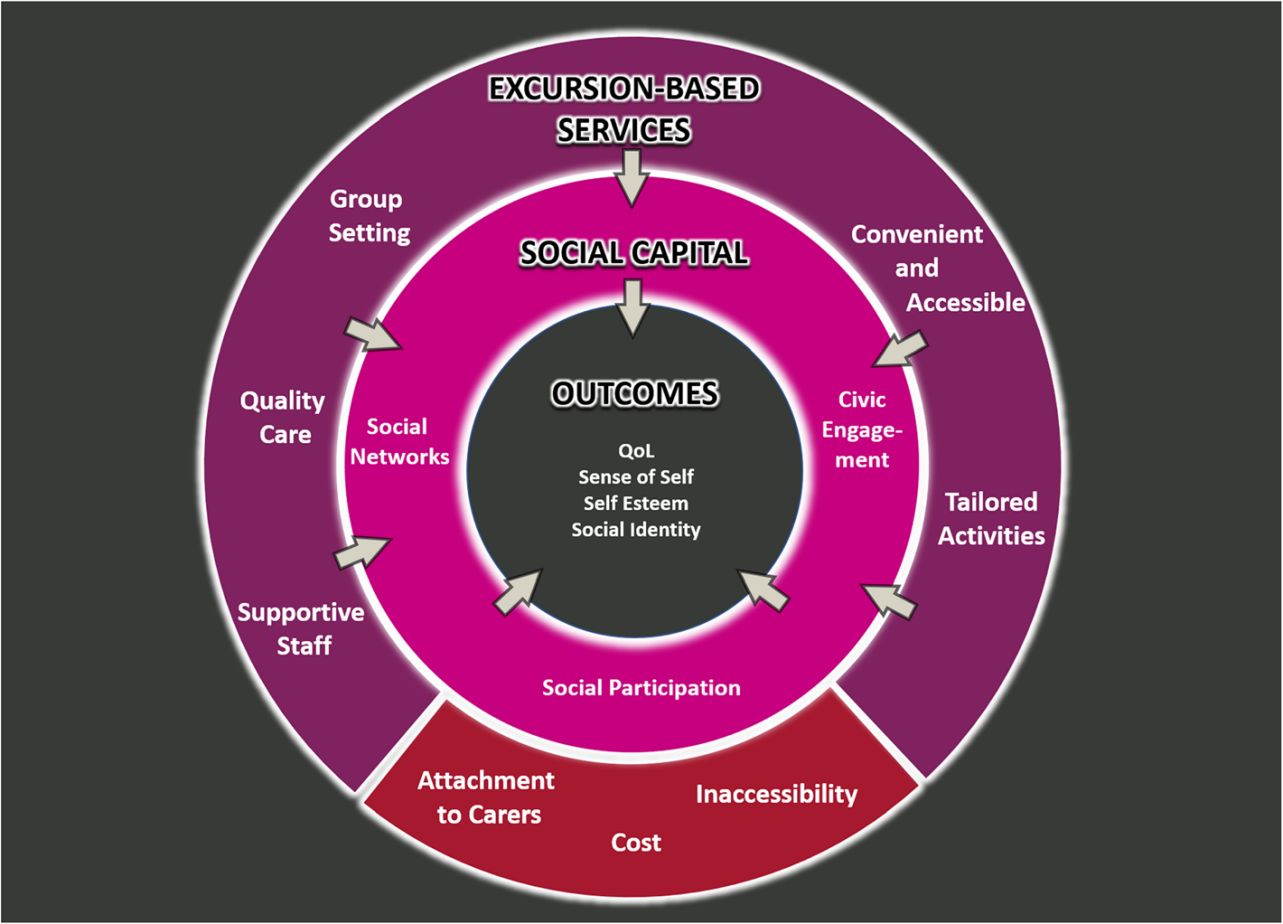
Visual summary of the mechanisms in the program and its influence on individual social capital and outcomes including quality of life (QoL). Original image produced by the authors

Excursion-based programs can build social capital and improve older adults’ physical and social outcomes. Social capital theory posits that individual behaviours (e.g., community participation), norms (e.g., trust in community and reciprocity) and macrolevel mechanisms (e.g., historical, political, and economic aspects) can affect health status [8, 40]. The aim of the Community Connections program was to build community networks and participation, and opportunities for exchange, and results indicate that this contributed to maintaining participants’ social identity, confidence, quality of life, and sense of self-worth (see Fig. 3*).* Concepts of bonding and bridging social capital are also highlighted in our findings. Bonding social capital refers to a close trusting relationship between a network of individuals who share a similar identity while bridging social capital is the relationship between people and groups who are not alike [41]. Being in a group setting and having shared interests with a common goal over a period of time was crucial in expanding the quality and quantity of bonding social capital among newfound friends, which had an impact on individual outcomes.

To enable vulnerable older adults to build social connections, it is important for social programs to recognize both their basic physical and social needs. Further, when programs help to enlarge the social space for older adults, they should focus on having universal access to the location or activity. As identified by the stakeholders, transportation and convenience were strong factors that drove the success of program. Carers particularly valued the supplied pick-up and drop-off services. Accessibility and flexibility are primary drivers of successful social engagement of ADS programs [42]. Providing suitable transportation, particularly in regional areas, will assist in enabling older adults to attend social participatory activities [43]. Aging policies and future community initiatives targeting isolated older adults should therefore ensure adequate transportation services are available and known. Future programs should also advocate for and engage the larger community to enhance the social capital at the community level and contribute to the common wellbeing of the community.

### Strengths and limitations

The use of mixed-methods, which involved combining a before and after survey analysed quantitatively with a qualitative exploration through semi-structured interviews and thematic analysis, was a strength of the current study. It allowed for the examination of not only the association between becoming a member of a community group on quality of life over an extended period, but also obtained a deeper understanding of the underlying reasons behind these associations.

The results of this study were limited by several factors. Our small sample size means that our findings may not be generalisable to the wider population of Australian community-based aged care users. The small sample size also precluded examining the impact of the program on subgroups (e.g. by gender, ethnicity) who may experience the benefits and challenges of excursion group-based programs differently. Furthermore, whilst the assessments were self-completed, participants may know the staff who provided the survey and may have resulted in assessor bias. We recommend future studies adopt quasi-experimental or randomised control designs to establish how different models and features of ADSs influence quality of life of community aged care older adults over a longer period of time. Future research is also required to explore barriers which may inhibit access to ADSs in Australia.

## Conclusion

Older people are at acute risk of experiencing social isolation and loneliness. Our results represent some of the first evidence of the effectiveness of excursion group-based activities to improve quality of life for community aged care individuals. Importantly the study identified some of the key elements of the program which appeared to be central to its success for older people and their carers. Aging policy and strategies should focus on initiatives that promote social connectivity with the wider community, to assist in reducing loneliness and improve outcomes for older adults.

## Supplementary Information

**Additional file 1: Figure S1.** Research flow chart. **Figure S2.** Mind map visualisation of the themes following a thematic framework for staff stakeholders. **Table S1.** Characteristics of participants engaged in the social program involved in the qualitative interviews. **Table S2.** Interview schedule with different stakeholders.

## Data Availability

The datasets used and/or analysed during the current study are available from the corresponding author on reasonable request.

## Abbreviations

ASCOT: Adult Social Care Outcomes Toolkit
ADSs: Adult day services
CHSP: Commonwealth Home Support Programme
HCP: Home Care Package

## References

[1] Ong AD, Uchino BN, Wethington E (2016). Loneliness and health in older adults: a mini-review and synthesis. Gerontology.

[2] Australian Institute of Health and Welfare. (2019). Social isolation and loneliness. Webpage. Canberra: AIHW. Retrieved 7, Jan 2020, from https://www.aihw.gov.au/reports/australias-welfare/social-isolation-and-loneliness

[3] Yang YC, Boen C, Gerken K, Li T, Schorpp K, Harris KM (2016). Social relationships and physiological determinants of longevity across the human life span. Proc Natl Acad Sci.

[4] Courtin E, Knapp M (2017). Social isolation, loneliness and health in old age: a scoping review. Health Soc Care Community.

[5] Muckenhuber J, Stronegger WJ, Freidl W (2013). Social capital affects the health of older people more strongly than that of younger people. Ageing Soc.

[6] Nyqvist F, Victor CR, Forsman AK, Cattan M (2016). The association between social capital and loneliness in different age groups: a population-based study in Western Finland. BMC Public Health.

[7] Bourdieu, P. (2018). The forms of capital. In: the sociology of economic life, third edition (pp. 78-92). Taylor and Francis

[8] Kawachi I, Subramanian SV, Kim D, Kawachi I, Subramanian SV, Kim D, SpringerLink (2008). Social capital and health.

[9] Australian Institute of Health and Welfare. (2013). The desire to age in place among older Australians. Bulletin no. 114. Cat. no. AUS 169. Canberra: AIHW. Retrieved 12, Jun 2019, from aihw.gov.au/getmedia/69a6b0b9-6f86-411c-b15d-943144296250/15141.pdf.aspx?inline=true

[10] Australian Institute of Health and Welfare. (2015). Australia’s welfare 2015. Australia’s welfare series no. 12. Cat. no. AUS 189. Canberra: AIHW. Retrieved 7, Jul 2019, from https://www.aihw.gov.au/getmedia/231c6801- 9956-46b4-af2c-7c778290122c/AW15–6-3-Older-Australians- and-the-use-of-aged-care.pdf.aspx

[11] Department of Health. (2019). CHSP interaction with home care packages – factsheet. Retrieved 14, Jan 2020, from https://agedcare.health.gov.au/sites/default/files/documents/02_2019/chsp_interaction_with_home_care_packages.pdf

[12] Brodaty H, Arasaratnam C (2012). Meta-analysis of nonpharmacological interventions for neuropsychiatric symptoms of dementia. Am J Psychiatr.

[13] Chen Y-RR, Schulz PJ (2016). The effect of information communication technology interventions on reducing social isolation in the elderly: a systematic review. J Med Internet Res.

[14] Atkins J, Naismith SL, Luscombe GM, Hickie IB (2013). Psychological distress and quality of life in older persons: relative contributions of fixed and modifiable risk factors. BMC Psychiatry.

[15] Jorgensen M, Siette J, Georgiou A, Warland A, Westbrook J. Modeling the association between home care service use and entry into residential aged care: a cohort study using routinely collected data. Journal of the American medical directors association, 19(2), 117-121.e3. 2018;19(2):117–121.e3. 10.1016/j.jamda.2017.08.004. 10.1016/j.jamda.2017.08.004 28951018

[16] Fields NL, Anderson KA, Dabelko-Schoeny H (2014). The effectiveness of adult day Services for Older Adults: a review of the literature from 2000 to 2011. J Appl Gerontol.

[17] Gardiner C, Geldenhuys G, Gott M (2018). Interventions to reduce social isolation and loneliness among older people: an integrative review. Health & Social Care in the Community.

[18] Milligan C, Neary D, Payne S, Hanratty B, Irwin P, Dowrick C (2016). Older men and social activity: a scoping review of Men’s sheds and other gendered interventions. Ageing Soc.

[19] Creswell JW, Plano-Clark VL (2007). Designing and conducting mixed methods research.

[20] Enrich Living Services. (2019). Community Connections. Retrieved 14, Jan 2020, from https://www.enrichliving.com.au/community-connections/

[21] Netten A, Beadle-Brown J, Caiels J, Forder JE, Malley J, Smith N (2011). ASCOT adult social care outcomes toolkit: Main guidance v2. 1 PSSRU Discussion Paper 2716/3.

[22] Cardona B (2018). Measuring outcomes of community aged care programs: challenges, opportunities and the Australian community outcomes measurement ACCOM tool. Health Qual Life Outcomes.

[23] Malley JN, Towers A-M, Netten AP, Brazier JE, Forder JE, Flynn T (2012). An assessment of the construct validity of the ASCOT measure of social care-related quality of life with older people. Health Qual Life Outcomes.

[24] Ritchie J, Spencer L (1994). Qualitative data analysis for applied policy research analysing qualitative data (pp.173–194).

[25] Khadka J, Lang C, Ratcliffe J, Corlis M, Wesselingh S, Whitehead C, Inacio M (2019). Trends in the utilisation of aged care services in Australia, 2008–2016. BMC Geriatr.

[26] Bruin SRD, Oosting SJ, Kuin Y, Hoefnagels ECM, Blauw YH, Groot LCPGMD, Schols JMGA (2009). Green care farms promote activity among elderly people with dementia. J Hous Elder.

[27] Jarrott SE, Gigliotti CM (2010). Comparing responses to horticultural-based and traditional activities in dementia care programs. Am J Alzheimers Dis Other Dement.

[28] Van Malderen L, Mets T, Gorus E (2013). Interventions to enhance the quality of life of older people in residential long-term care: a systematic review. Ageing Res Rev.

[29] Mikkelsen ASB, Petersen S, Dragsted AC, Kristiansen M. Social interventions targeting social relations among older people at nursing homes: a qualitative synthesized systematic review. Inquiry: a journal of medical care organization, provision and financing, 56. 2019;56:004695801882392. 10.1177/0046958018823929. 10.1177/0046958018823929 PMC6376508 30791836

[30] Dickens AP, Richards SH, Greaves CJ, Campbell JL (2011). Interventions targeting social isolation in older people: a systematic review. BMC Public Health.

[31] Bøen H. Characteristics of senior Centre users – and the impact of a group programme on social support and late-life depression. Norsk Epidemiologi. 2012;22(2). 10.5324/nje.v22i2.1574.

[32] Hutchinson SL, Gallant KA (2016). Can senior Centres be contexts for aging in third places. J Leis Res.

[33] Golding BG (2011). Social, local, and situated: recent findings about the effectiveness of older Men’s informal learning in community contexts. Adult Educ Q.

[34] Haslam C, Cruwys T, Milne M, Kan C-H, Haslam SA (2016). Group ties protect cognitive health by promoting social identification and social support. J Aging Health.

[35] Merriam SB, Kee Y (2014). Promoting community wellbeing: the case for lifelong learning for older adults. Adult Educ Q.

[36] Hikichi H, Kondo N, Kondo K, Aida J, Takeda T, Kawachi I (2015). Effect of a community intervention programme promoting social interactions on functional disability prevention for older adults: propensity score matching and instrumental variable analyses, JAGES Taketoyo study. J Epidemiol Community Health.

[37] Hikichi H, Kondo K, Takeda T, Kawachi I (2017). Social interaction and cognitive decline: results of a 7-year community intervention. Alzheimers Dementia.

[38] Franck L, Molyneux N, Parkinson L (2016). Systematic review of interventions addressing social isolation and depression in aged care clients. Qual Life Res.

[39] Grenade L, Boldy D (2008). Social isolation and loneliness among older people: issues and future challenges in community and residential settings. Aust Health Rev.

[40] Pollack CE, von dem Knesebeck O (2004). Social capital and health among the aged: comparisons between the United States and Germany. Health Place.

[41] Haslam SA, Jetten J, Postmes T, Haslam C (2009). Social identity, health and well-being: an emerging agenda for applied psychology. Appl Psychol.

[42] Millsteed J, Marquis R, Richmond J. The role of adult day Services in Supporting the occupational participation of people with dementia and their Carers: an integrative review. Healthcare. 2018;6(2). 10.3390/healthcare6020043. 10.3390/healthcare6020043 PMC6023311 29738489

[43] Tse T, Linsey H (2005). Adult day groups: addressing older people’s needs for activity and companionship. Australas J Ageing.

