# Supplementary material for: A mixed-methods study evaluating the impact of an excursion-based social group on quality of life of older adults
Source: BMC Geriatr. 2021 Jun 10;21:356. doi: 10.1186/s12877-021-02295-7 (PMC8194178; doi:10.1186/s12877-021-02295-7)
Supplement: Supplementary file 1 — Additional file 1: Figure S1. Research flow chart. Figure S2. Mind map visualisation of the themes following a thematic framework for staff stakeholders. Table S1. Characteristics of participants engaged in the social program involved in the qualitative interviews. Table S2. Interview schedule with different stakeholders. [file 12877_2021_2295_MOESM1_ESM.docx]

**Supplementary Material**

**Supplementary Figure 1.** Research flow chart

**Supplementary Figure 2.** Mind map visualisation of the themes following a thematic framework for staff stakeholders

**
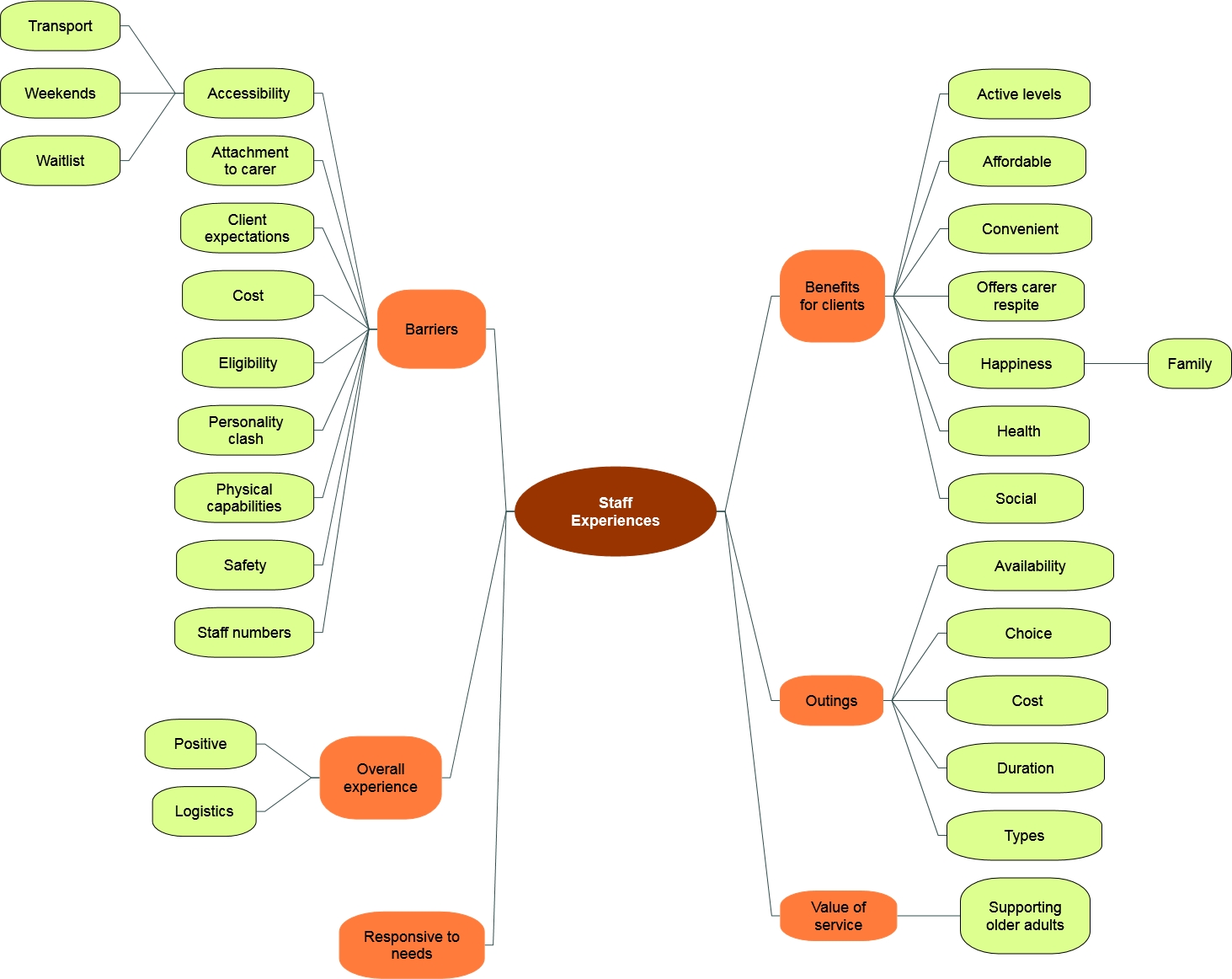
**

**Supplementary Table 1.** Characteristics of participants engaged in the social program involved in the qualitative interviews.

| Age Range | Gender | Range of use of Enrich Services | Number of outings (/month) | Types of outings completed |
| --- | --- | --- | --- | --- |
| 68-91 | 63.6% female | 2 months – 12 years | **2 – 12** | Whale watching  Helicopter ride  Harley Davidson ride  Pubs  Restaurants  Zoo  Railway launch  Yacht club  National park  Cinema  Concert  Boat ride  Camel ride  Vineyards  Art gallery  Cinema |

**Supplementary Table 2.** Interview schedule with different stakeholders.

| Stakeholder groups | Questions |
| --- | --- |
| Clients | 1. What was your experience of the Community Connections Program?    1. Was it acceptable to you? 2. Can you discuss the availability of the outings and the choice of outings? 3. Did you feel that Community Connections was responsive to your needs? 4. What are the perceived benefits for you? 5. What do you think the advantages or disadvantages were by attending Community Connections? 6. What was the value of the service for you? 7. What could be improved with the current program? |
| Staff | 1. What was your experience of the Community Connections Program?    1. How feasible was it to set up Community Connections?    2. Was it acceptable to your clients? 2. Can you discuss the availability of the outings and the choice of outings? 3. Did you feel that Community Connections was responsive to your client needs? 4. What are the perceived benefits for your clients? 5. What do you think the advantages or disadvantages were for clients who attended Community Connections? 6. What was the value of the service for you? 7. Were there any barriers in implementing Community Connections? |
| Family caregivers | 1. What was your experience of the Community Connections Program? 2. Did you feel that Community Connections was responsive to your client needs? 3. What are the perceived benefits for your cared one? 4. What are the perceived benefits for you? 5. What do you think the advantages or disadvantages were for your cared one? 6. What was the value of the service for you? 7. How can Community Connections be improved to address both your cared one and your needs? |

**Supplementary Table 3.** Initial thematic framework.

| **Role** | **Major theme** | **Sub-themes** |
| --- | --- | --- |
| Client | Overall perception | Acceptability  Gratefulness  Cost |
|  | Strength of program | Transport  Carers/Enrich staff – care, take suggestions on board  Outing quality and type  Organisation of outings |
|  | Suggested improvements | Length of outing  Targeting all client needs  Frequency and booking  Car/transport |
|  | Change/Benefit | Physical  Social – established friendships  Psychological – smiling all the time now |
|  | Feasibility | Awareness  Booking  Attending |
|  | Needs | Physical – got to get out of the house  Social – don’t have anyone to talk to |
| Staff | Feasibility | Establishing program  Organising schedule  Informing clients |
|  | Advantages | Social  Physical |
|  | Disadvantages | Mobility |
|  | Personal value | Contributing to client’s wellbeing |
|  | Barriers to implementation | Welfare  Safety  Location  Timing  Staff |
|  | A powerful experience | Making a difference matters  Emotional involvement  Learning? |
| Carer | Advantage | Cared for appropriately (transport and staff)  Time off/respite  Adding value to their cared one’s life |
|  | Limitations | Cost – limiting outing frequency |
|  | Initial approach | Scepticism  Collaborative approach |
|  | Change in cared one | Positive; psychological |
|  | Suggestions | Online booking |
